# A deep learning-based micro-CT image analysis pipeline for nondestructive quantification of the maize kernel internal structure

**DOI:** 10.1016/j.plaphe.2025.100022

**Published:** 2025-02-28

**Authors:** Juan Wang, Si Yang, Chuanyu Wang, Weiliang Wen, Ying Zhang, Gui Liu, Jingyi Li, Xinyu Guo, Chunjiang Zhao

**Affiliations:** a College of Information, Shanghai Ocean University, Shanghai, 201306, China; b Information Technology Research Center, Beijing Academy of Agriculture and Forestry Sciences, Beijing, 100097, China; c Beijing Key Lab of Digital Plants, National Engineering Research Center for Information Technology in Agriculture, Beijing, 100097, China; d College of Computer Science and Engineering, Southwest Minzu University, Chengdu, Sichuan, 610225, China

**Keywords:** Maize kernel, Vitreous endosperm, Starchy endosperm, Semantic segmentation, Mirco-CT

## Abstract

Identifying and segmenting the vitreous and starchy endosperm of maize kernels is essential for texture analysis. However, the complex internal structure of maize kernels presents several challenges. In CT (computed tomography) images, the pixel intensity differences between the vitreous and starchy endosperm regions in maize kernel CT images are not distinct, potentially leading to low segmentation accuracy or oversegmentation. Moreover, the blurred edges between the vitreous and starchy endosperm make segmentation difficult, often resulting in jagged segmentation outcomes. We propose a deep learning-based CT image analysis pipeline to examine the internal structure of maize seeds. First, CT images are acquired using a multislice CT scanner. To improve the efficiency of maize kernel CT imaging, a batch scanning method is used. Individual kernels are accurately segmented from batch-scanned CT images using the Canny algorithm. Second, we modify the conventional architecture for high-quality segmentation of the vitreous and starchy endosperm in maize kernels. The conventional U-Net is modified by integrating the CBAM (convolutional block attention module) mechanism in the encoder and the SE (squeeze-and-excitation attention) mechanism in the decoder, as well as by using the focal-Tversky loss function instead of the Dice loss, and the boundary smoothing term is weighted as an additional loss term, named CSFTU-Net. The experimental results show that the CSFTU-Net model significantly improves the ability of segmenting vitreous and starchy endosperm. Finally, a segmented mask-based method is proposed to extract phenotype parameters of maize kernel texture, including the volume of the kernel (V), volume of the vitreous endosperm (VV), volume of starchy endosperm (SV), and ratios over their respective total kernel volumes (VV/V and SV/V). The proposed pipeline facilitates the nondestructive quantification of the internal structure of maize kernels, offering valuable insights for maize breeding and processing.

## Introduction

**Fig. 1:**
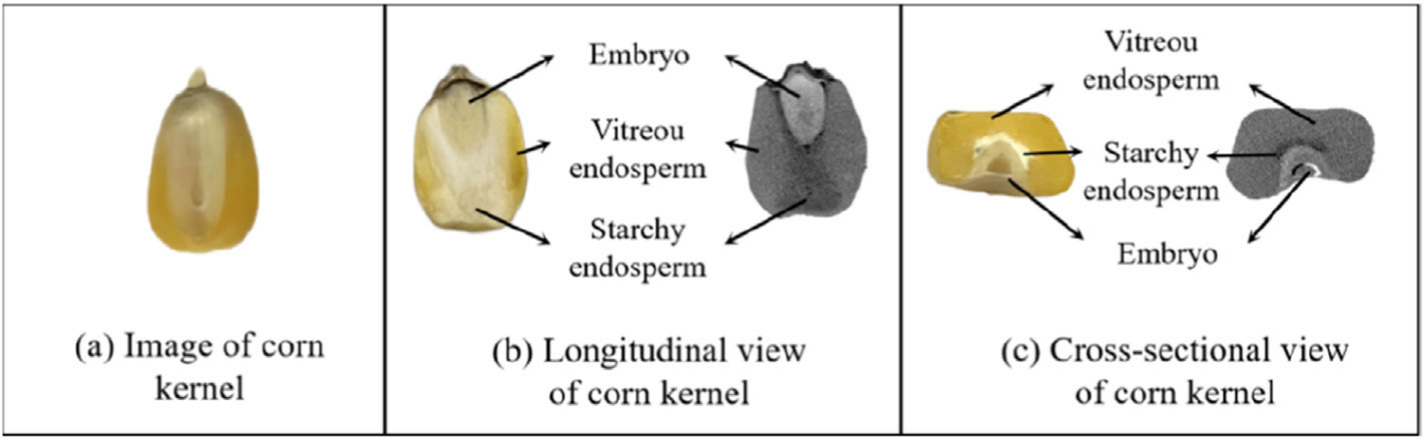
Morphological and cross-sectional images of a maize kernel. (a) Morphological diagram of a maize kernel. (b) Longitudinal section diagram and CT image. (c) Maize grain transverse section diagram and CT image.

Crops such as maize are cultivated around the world and are used for a variety of purposes, including feed, food, and chemicals. In recent decades, research on maize has developed rapidly [1]. Maize kernels are composed of three main organs: the germ, endosperm and pericarp [2]. Endosperm accounts for approximately 90% of the dry weight of maize kernels and largely affects the grain weight and nutritional quality of maize kernels [3]. The internal structural phenotype of maize grains is an important agronomic trait of maize. It is influenced by the ratio of vitreous endosperm to starchy endosperm within the maize grain. In the endosperm, the vitreous region is typically located at the perimeter of the endosperm and is glassy and translucent, whereas the starchy endosperm is typically located at the center and is white and powdery [4]. The vitreous endosperm strengthens and safeguards grains from mechanical damage during harvest and transportation, whereas the starchy endosperm remains fragile and vulnerable to pests and diseases [5]. Therefore, it is particularly important to research the phenotypic characteristics of maize kernels in detail. Fig. 1(a) shows the external morphology of the maize kernel. Fig. 1(b) and (c) depict the anatomical and CT scan images of different axes, respectively. The growth position and area of the embryo, starchy endosperm and vitreous endosperm can be clearly observed. Zhang et al. used the traditional method of researching the internal structure of maize kernels involves manual dissection or grain sectioning to observe the longitudinal cross section of the kernels [6]. To calculate the vitreous endosperm area as a percentage of the whole endosperm, Wang et al. [7] cut the grain horizontally and photographed it under an optical microscope stereoscope (LeicaM165FC). The percentage of vitreous endosperm was determined via ImageJ software to measure the seeds and calculate the relative value. To determine the grain structure, Guo et al. [8] immersed the grain for 20–36 ​h, divided the blade into a grain embryo, vitreous endosperm, starchy endosperm and other structures, and dried it to determine the dry weight. This approach is destructive, complex, inefficient, and provides limited phenotypic parameters, making it inadequate for modern maize breeding.

In recent years, deep learning technology has made significant progress in computer vision, especially in image segmentation and feature extraction. The successful application of these technologies has brought new opportunities for agricultural imaging, especially in crop phenotype analysis, disease detection and growth monitoring. Compared with traditional image processing methods, deep learning can effectively overcome the limitations of traditional methods in dealing with complex images through automatic feature extraction and pattern recognition. For example, in traditional threshold segmentation and region growing methods, accurate segmentation results are often difficult to obtain in the face of low-contrast images and complex boundaries [9]. The deep learning model, especially the convolutional neural network (CNN), can automatically identify and extract important features in an image through multilevel feature learning, thus significantly improving the accuracy of image segmentation [10]. In addition, deep learning has shown significant advantages in accelerating the analysis process and achieving nondestructive testing. Taking micro-CT technology as an example, combined with deep learning models, researchers can obtain high-resolution internal structure images without destroying samples. This nondestructive detection method provides an ideal basis for scientific agricultural research, especially when the internal structure of corn seeds is analyzed [11]. Through the combination of deep learning and micro-CT, we can capture the complex structural features inside a crop more accurately, thus providing more reliable data support for subsequent quantitative analysis [12]. Lurstwut and Pornpanomchai [13] employed digital image processing and artificial neural networks to analyze the appearance characteristics of rice seeds. Similarly, Qiao et al. proposed a quantitative hardness detection method based on hyperspectral imaging technology to achieve rapid and nondestructive detection of maize kernel hardness [14]. These studies use computer vision, visible light imaging, hyperspectral imaging and other technologies to obtain nondestructive phenotypic data outside the grain. Moreover, there are some deficiencies in obtaining the internal structure information of grains. Most of these methods can obtain only two-dimensional image data of grains. In many cases, however, three-dimensional models are needed to analyze grain traits [15,16].

In this context, micro-CT technology, as a nondestructive imaging method, can provide high-resolution internal structure images and provide an ideal basis for the quantitative analysis of crop internal characteristics. Combined with deep learning technology, micro-CT can effectively overcome the limitations of traditional imaging methods in dealing with complex structures and provide more accurate tools for scientific agricultural research. This nondestructive imaging technique visualizes and quantifies the internal structure of opaque objects at submicron to nanometer scales. This imaging method is extensively used in grain crop analysis. For example, Crozier et al. [17] extracted phenotype measurements from CT scans of sorghum grains. The embryo volume, ratio of vitreous to starchy endosperm, endosperm volume, pericarp volume, and kernel volume were measured. In research on tomato seed phenotypes, L. et al.used X-ray microchromatography to scan 105 tomato seeds and qualitatively and quantitatively determined the internal and external morphological traits, such as the solid phase volume, thickness and surface area of tomato seeds in three-dimensional space [18]. Similarly, Hu et al. [19] introduced an X-ray computed tomography-based image analysis method to extract 3D features of 22 rice grains for variety classification and yield evaluation. Alvarado et al. (2019) effectively utilized X-ray microcomputed tomography and image processing techniques to extract the morphological parameters of developing wheat grains, revealing significant characteristics of wheat grain growth. Currently, X-ray computed tomography technology is used to study maize kernels [20]. Gustin et al. [21] utilized X-ray micro-CT technology to measure the density, volume, and cavity volume of maize kernels. Guelpa et al. [22] utilized X-ray microcomputer tomography to construct a density calibration curve for corn grains, enabling the successful classification of maize grain hardness by analyzing the density and porosity of various parts. This method offers an effective classification technique for the dry grinding industry. Li et al. created an image-based automated method to evaluate the three-dimensional morphology of maize kernels, enabling rapid extraction and quantification of compositional and structural traits across different maize cultivars [23]. In research on the vitreous and starchy endosperm of maize kernels, Yin et al. [24] employed X-ray computed tomography to scan test samples and reconstructed the three-dimensional structure of maize kernels using image filtering and threshold segmentation. The structural parameters of the embryo, endosperm, subcutaneous cavity, embryo cavity, vitreous endosperm, starchy endosperm, and endosperm cavity were obtained for different kernel positions on the maize ear. Among them, the distinction between vitreous and starchy endosperm is a key step. However, the complexity and meticulous nature of this process result in a significant workload and are time-consuming. Recent studies have shown the potential of X-ray computed tomography (CT) for analyzing the internal structures of crops such as sorghum, wheat, and corn [25]. Du et al. [23] extracted numerous phenotype metrics both inside and outside maize kernels from CT images, and the results were consistent with manual measurements. Building on this, we further segmented the endosperm part of the maize kernels. There is still a lack of systematic literature reports on the intrinsic phenotypes of the vitreous endosperm and starchy endosperm of maize kernels. This research field is still in its infancy. Therefore, it is particularly important to study the methods for accurately identifying and effectively segmenting these two endosperm types.

There are abundant variations in the texture of maize grains in natural populations, ranging from almost completely vitreous to completely starchy materials [26]. Traditional techniques for detecting the internal structure of maize kernels usually require manual dissection or sectioning to observe longitudinal cross sections. These measures are destructive, time-consuming and tedious, and the phenotype parameters they extract are limited. In agricultural imaging, the existing segmentation methods include threshold-based segmentation, region growing methods, and traditional machine learning methods (such as support vector machines and random forests). These methods have achieved some success in some cases, but they often have limitations when dealing with low-contrast images and complex boundaries. For example, the threshold-based segmentation method is prone to oversegmentation or undersegmentation when the image contrast is insufficient, and the region growing method is sensitive to the selection of initial seed points, which may lead to unstable segmentation results. In addition, traditional machine learning methods usually rely on manual feature extraction, which has difficulty adapting to the complex internal structure of crops. Deep learning technology has recently made significant advancements in image segmentation [27]. However, its application in agriculture, especially in the segmentation of the vitreous endosperm and starchy endosperm of maize kernel CT images, is relatively rare.

In view of these limitations, our proposed CSFTU-Net model significantly improves the segmentation accuracy of the internal structure of maize seeds by introducing an attention mechanism and an improved loss function. The following summarizes our contributions.

1) To address the issue of low contrast and indistinct boundaries between vitreous and starchy endosperm in maize kernels, we employ image processing methods, such as contrast enhancement and pixel truncation. We enhance the U-Net segmentation network by integrating the CBAM (convolutional block attention module) into each decoder layer and the SE (squeeze-and-excitation attention) module into each encoder layer for training. The focal Tversky loss function optimizes image quality for the accurate recognition and segmentation of vitreous and starchy endosperm.
2) The proposed CSFTU-Net method is comprehensively evaluated, and a Dice score of 89.13 % is achieved on the test set of 1000 CT datasets, which exceeds those of other common segmentation methods.
3) According to the segmentation results, the phenotype parameters related to the internal structure of maize grains, such as the vitreous endosperm volume, starchy endosperm volume, vitreous and starchy endosperm volume ratios of the grain population and different subpopulations, are extracted, which provides a valuable reference for maize breeding and processing.

## Materials

### Data acquisition

**Fig. 2:**
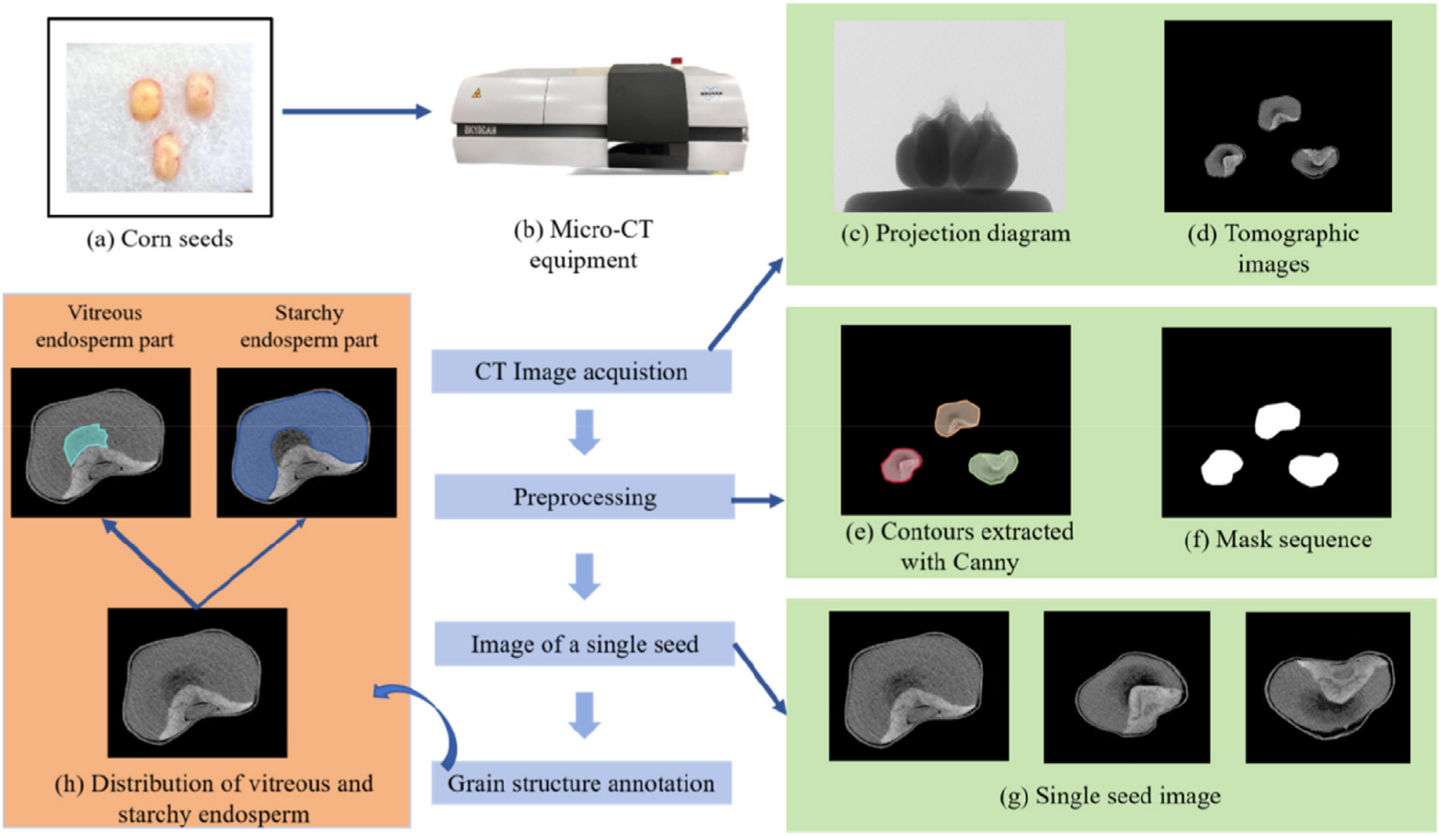
Micro-CT image acquisition and preprocessing. (a) Three seeds per variety. (b) Scanning of seeds by CT with the SKYSCAN 1273 model. (c) Projected images of maize kernels generated after scanning. (d) Seed tomography images obtained by processing the raw scans using NRecon software. (c)–(g) Splitting of three seeds in the tomogram. (h) Structure of the vitreous and starchy endosperm.

The experiment utilized 250 varieties of maize seeds sown in Hainan Sanya by the Beijing Academy of Agricultural and Forestry Sciences in 2018. The materials are categorized into four subgroups: mixed (mixed germplasm), NSS (nonhard germplasm), SS (hard germplasm), and TST (tropical/subtropical germplasm). Three corn kernels with no surface damage are selected for each variety, and CT images are obtained by scanning with a Bruker 1172 micro-CT system (USA) (Fig. 2 (a)). The scanning parameters used in this experiment are shown in Table 1.

**Table 1:** Micro-CT scanning and reconstruction parameters.

Parameters	Numeric
Input voltage/kV	40
Amps/μA	250
Single rotation angle/(°)	0.4
Total Rotation angle/(°)	180
Resolution/μm	13.55
Distance between the sample and light source/mm	259.850
Distance between the sample and light source/mm	345.591

A batch scanning method is employed to increase the efficiency of corn grain CT imaging. For each scan, three grains of one variety are fixed on a foam board using double-sided glue and then placed in a CT device for scanning (Fig. 2(a)-(b)). After the micro-CT scan is completed, a projection image of the grains is obtained (Fig. 2(c)). The original image from the CT scan is reconstructed by CT Scan NRecon (version 1.6.9.4) software to obtain a series of reconstructed virtual images in 8-bit BMP cross-sectional format (Fig. 2(d)). A total of 250 samples are collected, yielding 30,000 original CT images, each with dimensions of 2000 ​× ​2000 pixels.

### Image preprocessing and data annotation

The CT image contains three grains. In this work, the edge-based segmentation method is used to directly extract the CT image of a single maize kernel (Fig. 2(e)-(g)) using the Canny edge detector (Wang et al., 2024). To facilitate data labeling, this paper uses the dcm2niix toolkit to convert the dicom file into nii.gz format data. The distributions of vitreous and starchy endosperm components in maize kernels in CT images are shown in Fig. 2(h).

**Fig. 3:**
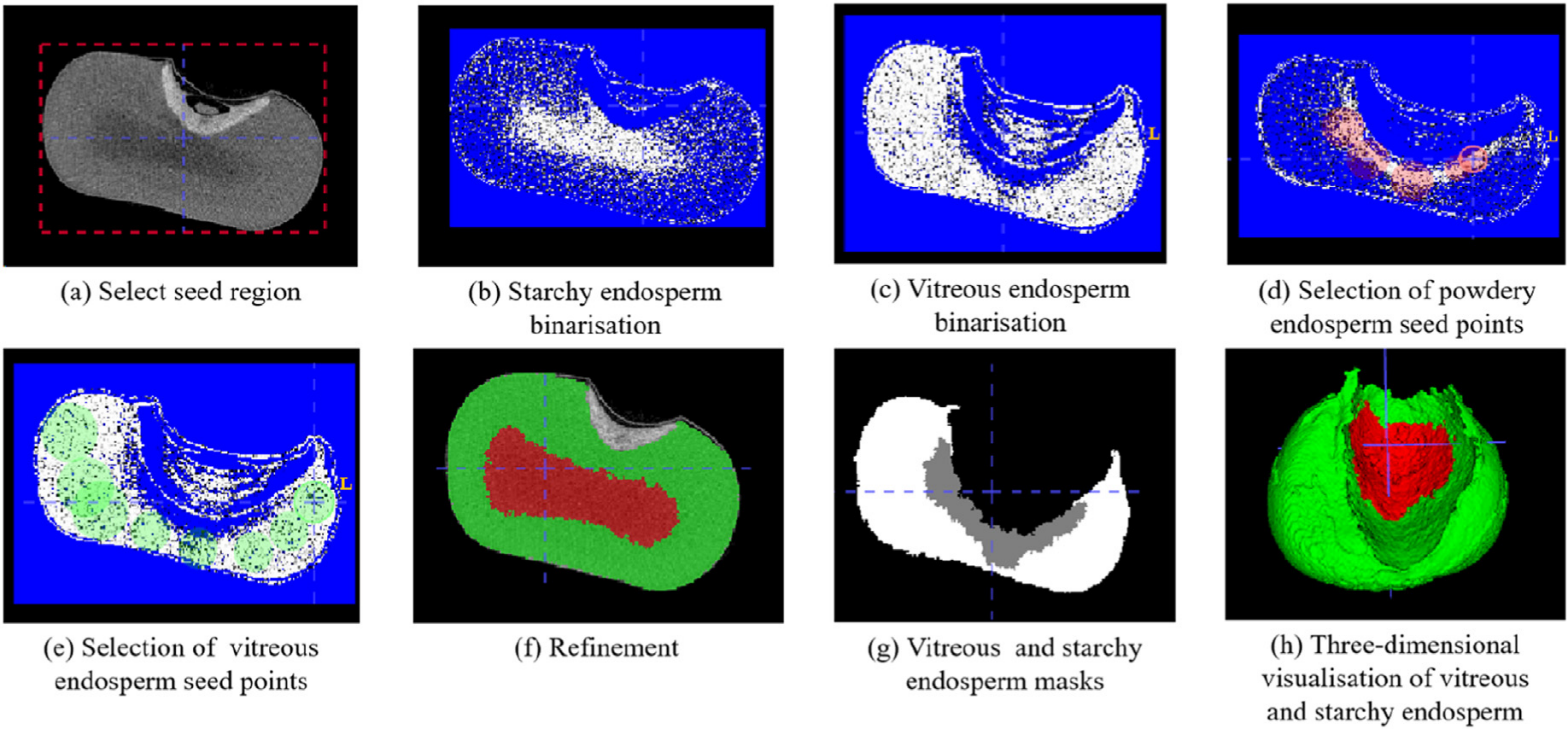
Process of labeling data using ITK-SANP. (a) Original image. (b)–(f) Specific step in data labeling. (g) Masked map of labeled results. (h) Labeling of a complete seed grain after three-dimensional reconstruction of the map.

The open-source software ITK-SNAP is used to manually and interactively label vitreous and starchy endosperm in maize kernel images. The process of labeling is as follows: first, the region where the grain is located (the dotted line frame in Fig. 3(a)) is manually selected, and the binarization threshold is adjusted to initially extract the vitreous endosperm and starchy endosperm (Fig. 3(b)-(c)); then, the seed points are manually selected for regional growth (Fig. 3(d)-(e)), and the brush and morphological tools can be used to finely adjust the mask layer by layer (Fig. 3(f)). Finally, the labels of vitreous endosperm and starchy endosperm corresponding to the CT images are derived (Fig. 3(g)). Three-dimensional visualization of the vitreous endosperm and starchy endosperm is shown in Fig. 3(h).

**Fig. 4:**
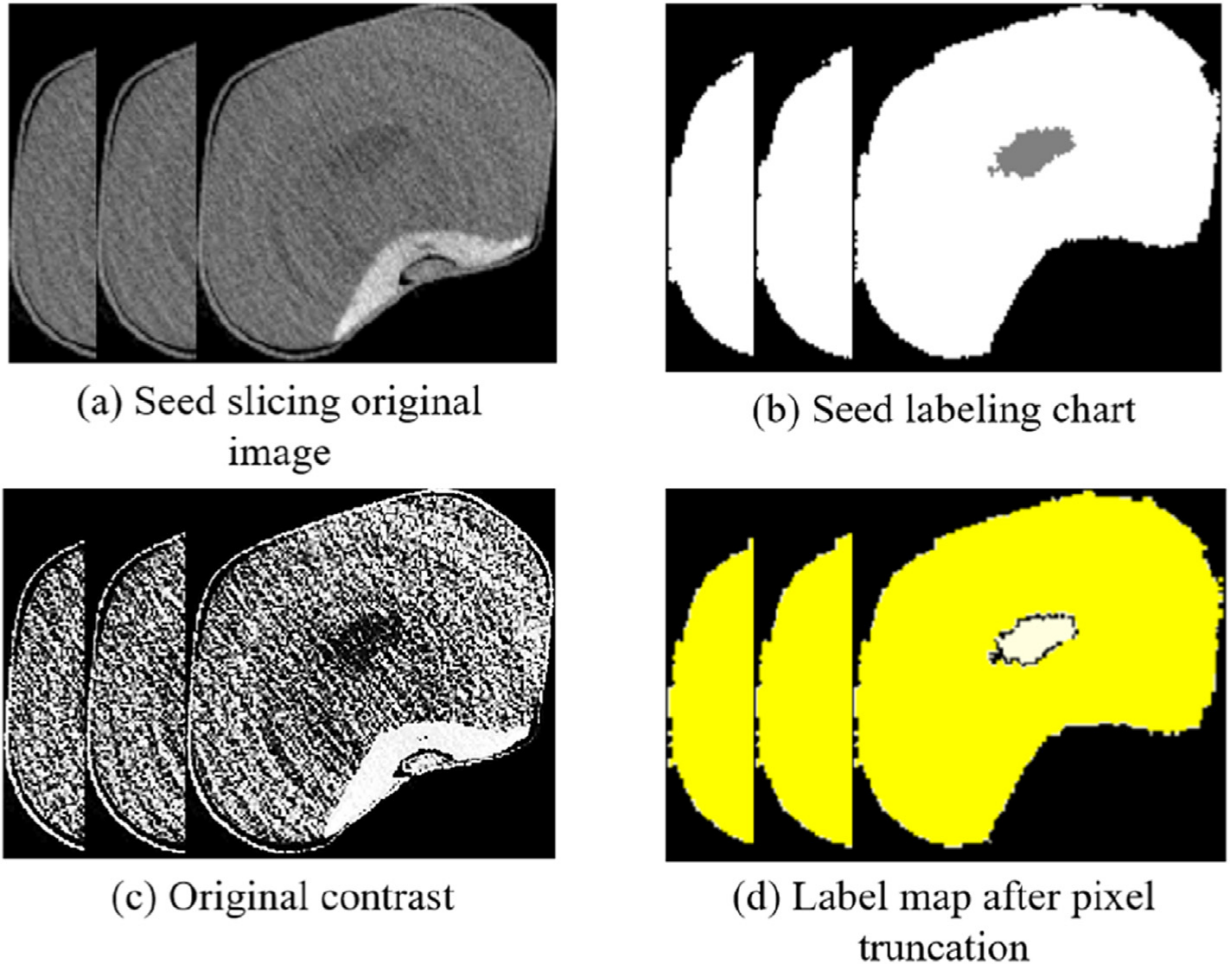
Data processing. (a) Tomogram of the corn kernels. (b) Maize kernel labeling diagram. (c) Vitreous and starchy endosperm portions of the original image that are contrast-enhanced. (d) Image generated after pixel truncation of the labeled figure. The white part is the starchy endosperm, and the yellow part is the vitreous endosperm.

ITK-SNAP is used to export the labeled grain data in.nii format, and python code is used to convert the.nii data into the.png format. A total of 30,000 grain slices and corresponding label maps are obtained (Fig. 4(a)-(b)). After conversion, the image resolution is 256 ​× ​256, and the bit depth is 24. To improve the computational efficiency of the model, the bit depth of the label graph is changed to 8. To increase the edge sharpness of the cross-sectional images of the vitreous endosperm and starchy endosperm, the contrast of the original image of the grain section is increased (Fig. 4(c)). In addition, because the dataset is large, to avoid gradient explosion and disappearance, the label map is truncated by pixel values. The pixel value of the starchy endosperm label image is truncated to 1, the pixel value of the vitreous endosperm is truncated to 2, and the background pixel value of 0 is unchanged (Fig. 4(d)). Finally, the training and validation sets are assessed at a ratio of 8:1, and the remaining 3000 samples are used in the test sets to assess the segmentation performance. The formula for pixel truncation is as follows:

(1) $$P^{\prime }=\left\{ \begin{array}{c} \min\_val\;if\;P\;<\;\min\_val\;\\ \max\_val\;if\;P\;>\;\max\_val\\ P\;Otherwise \end{array}\right.$$

where *P* is the original pixel value and where $P^{\prime }$ is the truncated pixel value. min_val and max_val are the set minimum and maximum thresholds, respectively. In this work, min_val is set to 0, max_val is set to 255, and *P* is set to 128.

## Method

### Model for the segmentation of the maize kernel endosperm

U-Net is a popular convolutional neural network originally designed for medical image segmentation [28]. Its structure consists of a contraction path and an expansion path, and each path contains multiple convolution and pooling layers. In the shrinking path, the network reduces the image’s spatial dimensions through successive convolution and pooling layers while increasing feature channels to capture contextual information. The extended path incrementally restores the image’s spatial resolution and details via deconvolution, integrating the feature map with corresponding layers in the shrinkage path. This process enhances the network’s utilization of location information in the output layer. This U-Net structure is particularly suitable for processing images with complex backgrounds and irregular objects, so it is widely used in medical image processing, such as for cell segmentation and tumor detection [[29], [30], [31]].

#### *CSFTU-net segmentation models*

The CSFTU-Net model is an improved version of the conventional U-Net architecture that aims to significantly improve the accuracy and robustness of image segmentation tasks. The model architecture is composed of two main parts: an encoder and a decoder. In the encoder part, the model uses multilayer convolution and pooling operations to gradually extract image features. These convolutional layers are often followed by batch normalization and ReLU activation functions to enhance the nonlinear expression ability of the model. With the advancement of the downsampling step, the spatial resolution of the feature map gradually decreases, whereas the number of channels increases accordingly, thus effectively capturing the high-level features of the image. The decoder part gradually restores the spatial resolution of the image through the upsampling layer (as in deconvolution or interpolation methods). In this process, the model combines feature maps from the encoder (via skip connections) to preserve and fuse the details of the image. After each upsampling step, the convolution layer is used to further process the feature map to ensure that the final output resolution is consistent with the input image.

To further enhance the model’s ability to focus on important features, CSFTU-Net integrates the CBAM and the SE mechanisms. The CBAM weights the feature map through the channel attention and spatial attention modules [32], whereas the SE module enhances the importance of features by adaptively recalibrating the feature channels [33]. In the model output layer, the 1 ​× ​1 convolutional layer is used to map the feature map to the number of target categories, thereby outputting the final segmentation result.

In terms of parameter settings, CSFTU-Net uses well-designed hyperparameters. The initial learning rate is set to 0.0001, the minimum learning rate is set to 0.00001, and the RMSprop optimizer is used for training. The batch size is set to 1 to adapt to memory constraints and improve the training stability of the model. The model is trained for 50 epochs to ensure sufficient learning of image features. In addition, the number of filters in the convolution layer can be adjusted according to the complexity of the task, usually between 64 and 512, and the convolution kernel size is usually maintained at 33 to effectively preserve the spatial information of the feature map. In summary, the CSFTU-Net model achieves high performance in image segmentation tasks by optimizing architecture design and parameter settings.

#### *CBAM module and SE module*

**Fig. 5:**
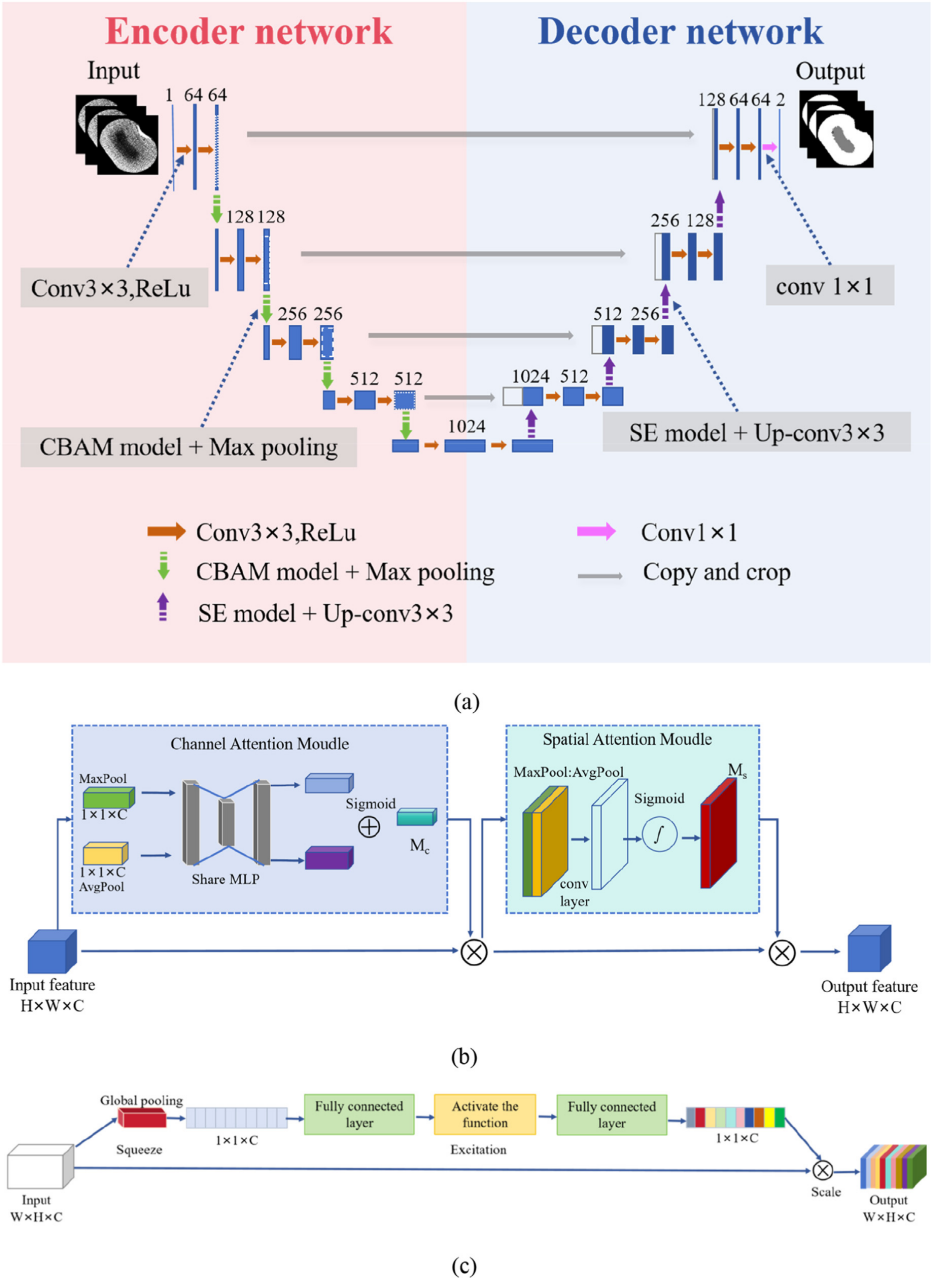
(a) CSFSU-Net structure diagram. The CBAM mechanism is added in each downsampling layer, and the SE mechanism is added in each upsampling layer at U-Net. (b) CBAM structure. (c) Channel attention and spatial attention modules of SE.

The network is divided into five layers, and four downsampling operations are performed in the coding stage. During each downsampling process, the CBAM is embedded in front of the maximum pooling layer, which can adjust the multiple channels and ensure that the graph is not too small to cause overfitting. The CBAM is embedded before each maximum pooling operation to modulate multichannel features and prevent excessive reduction in image dimensions, thereby reducing the risk of overfitting (Fig. 5(b)). Initially, the processes the input feature map F (W ​× ​H ​× ​C) through global max pooling and global average pooling along the width and height, resulting in two 1 ​× ​1 ​× ​C feature maps. These are then passed through a shared two-layer neural network (MLP). The first layer has C/r neurons (with ReLu activation), and the second layer has C neurons. The MLP output is elementwise combined and activated by a sigmoid function to produce the channel attention feature. The feature is multiplied elementwise with the input feature map F to generate the input features for the spatial attention module as follows:

(2) $$F_{avg}^{c}=\frac{1}{H\times W}\sum_{i=1}^{H}\sum_{j=1}^{W}F\left(c,i,j\right)$$

(3) $$F_{avg}^{c}=\max_{i,j}F\left(c,i,j\right)$$

(4) $$M_{C}=\sigma (MLP\left(AvgPool\left(F\right)\right)+MLP\left(MaxPool\left(F\right)\right)$$

where F(c,i,j) denotes the eigenvalue at channel c, location i, and j. Then, the two pooling results are sent to a shared network (usually including two fully connected layers) to generate weights. σ is a sigmoid function, and MLP is a multilayer perceptron.

The spatial attention module receives the feature map F from the channel attention module. Initially, global max pooling and average pooling operations are applied along the channel dimension, producing two H ​× ​W ​× ​1 feature maps. These maps are concatenated along the channel axis and followed by a 7 ​× ​7 convolutional layer that compresses them to a single H ​× ​W ​× ​1 channel. The resulting spatial attention map is then computed using a sigmoid activation. Finally, the spatial attention feature is multiplied elementwise with the original input feature map to generate the final output as follows:

(5) $$F_{spatial}^{avg}=\frac{1}{C}\sum_{c=1}^{C}F\left(c,i,j\right)$$

(6) $$F_{spatial}^{\max}=\max_{c}\sum_{c=1}^{C}F\left(c,i,j\right)$$

(7) $$M_{S}=\sigma \left(f^{7\times 7}\left(\left[F_{spatial}^{avg};F_{spatial}^{\max}\right]\right)\right)$$

where $F_{spatial}^{avg}$ and $F_{spatial}^{\max}$ are stitched together and the spatial attention map is generated through the convolution layer. $f^{7\times 7}$ represents a 7 ​× ​7 convolution filter, σ is a sigmoid function, and ‘;’ represents the splicing operation on the channel. The final output feature maps *F′* and F are obtained by multiplying the input feature map F with the two attention maps (channel and space) by the elements.

(8) $$F^{\prime }=\left(F⊗M_{C}\right)⊗M_{S}$$

where ⊗ represents multiplication by the element.

In the decoding stage, four upsamplings operation are performed, and the SE module (Fig. 5(c)) is embedded in each upsampling process to enhance the feature representation. The squeeze component compresses global spatial information into a channel descriptor via global average pooling. This reduces the W ​× ​H ​× ​C feature map to a 1 ​× ​1 ​× ​C vector, consolidating the channel features. The resulting channel-level statistical data capture context and mitigate channel dependence as follows:

(9) $$Z_{c}=F_{sq}\left(U_{c}\right)=\frac{1}{H\times W}\sum_{i=1}^{H}\sum_{j=1}^{W}u_{c}\left(i,j\right)$$

To utilize the information from the squeeze operation, the excitation operation captures channel dependence. The initial fully connected layer reduces the number of channels from C to C/r, and ReLU activation follows. The second fully connected layer restores the channels to C using weights derived from sigmoid activation. The final dimension is 1 ​× ​1 ​× ​C, representing the weight of C feature maps in U. Finally, the operation is used to weight the attention weights obtained above to the features of each channel. r is the compression ratio, and the formula is as follows:

(10) $$s=F_{ex}\left(z,W\right)=\sigma \left(g\left(z,W\right)\right)=\sigma \left(W_{2}\delta \left(W_{1}z\right)\right)$$

(11) $$x_{c}=F_{scale}\left(u_{c},s_{c}\right)=s_{c}u_{c}$$

#### *Focal Tversky loss function*

In maize endosperm segmentation, the fuzzy boundary problem is a crucial challenge that is directly related to the accuracy and reliability of the segmentation results. The causes of this problem are complex and diverse and mainly include the limitations of imaging technology, improper sample selection and processing, and the high complexity of the internal structure. Specifically, CT imaging technology may result in a significant reduction in the contrast between different parts of the endosperm due to insufficient scanning resolution or poor imaging conditions, which in turn causes blurred boundaries and increases the difficulty of model recognition and segmentation. In addition, the sample may be disturbed by environmental factors (such as temperature and humidity) during the imaging process, causing changes in physical properties and affecting the quality of CT images. The shortcomings of the image preprocessing stage, such as incomplete noise removal and insufficient image enhancement, also aggravate the boundary blur phenomenon. Furthermore, the complexity of the internal structure of the maize endosperm, especially in the junction area of the vitreous and starchy endosperm parts, is often accompanied by overlapping and mixing phenomena, which makes it difficult for the model to accurately identify and distinguish the boundaries of different regions during segmentation. Fuzzy boundaries may lead to excessive segmentation or insufficient segmentation of segmentation results, which in turn affects subsequent quantitative analysis and phenotypic parameter extraction but also increases the difficulty of model training, making it difficult for the model to capture key features effectively in the learning process and ultimately reducing the robustness and accuracy of segmentation.

To solve the fuzzy boundary problem, we choose the focal Tversky loss and introduce the boundary smoothing term as an additional loss term in the model. By applying smoothing constraints to the boundary region, the model can better capture the subtle differences between the endosperm. This strategy not only improves the accuracy of segmentation but also enhances the stability of the model in dealing with complex structures.

The focal Tversky loss [34] function combines the characteristics of focal loss [35] and Tversky loss [36]. It is a loss function designed to address the class imbalance problem. Its core goal is to focus the model’s attention on difficult-to-classify samples, thereby optimizing the overall performance of the model. By introducing three key parameters α (alpha), β (beta) and γ (gamma), the function achieves fine regulation of the importance of positive and negative samples, false-negative and false-positive penalties, and the impact of easily classified samples. The focal Tversky loss is calculated as follows:

(12) Loss=(1−Tversky)ˆγ×α

where Tversky is the result of the Tversky coefficient, which quantifies the similarity between two sets. In image segmentation tasks, this coefficient is derived from the overlap between the predicted outcomes and actual labels and is formulated as follows:

(13) $$Tversky=\frac{\left|X\cap Y\right|}{\left|X\right|\times \beta +\left|Y\right|\times \left(1-\beta \right)}$$

here, X is the prediction set, Y is the true set, and β is an adjustable parameter used to balance the weights of false positives and false-negatives. Specifically, the parameter α is set to control the weight of positive and negative samples in the loss calculation and increase the model’s emphasis on positive samples (or specific categories of concern). The parameter β is responsible for adjusting the contribution of false-negatives and false positives in the loss function and is set to 0.75. This setting makes the model more sensitive to difficult-to-classify samples (i.e., those that are easy to misjudge). The parameter γ is used to adjust the influence of easy-to-classify samples on the loss function, and the value is 2.0. By reducing the weight of easy-to-classify samples in the loss calculation, the model’s attention to difficult-to-classify samples is further increased.

In addition, we introduce the boundary smoothing term as an additional loss term and combine it with focal Tversky loss. This item is introduced to specifically solve the fuzzy boundary problem. By imposing smooth constraints on the boundary region, our model can better capture the subtle differences between the endosperm, thereby improving the accuracy and robustness of segmentation. In addition, we introduce the boundary smoothing term as an additional loss term and combine it with focal Tversky loss. The boundary smoothing term can be expressed as follows:

(14) $$L_{smooth}=\lambda \sum_{i,j}{\left(|\nabla I_{pred}\left(i,j\right)\left|-\right|\nabla I_{true}\left(i,j\right)|\right)}^{2}$$

where $I_{pred}$ and $I_{true}$ represent the segmentation map predicted by the model and the real segmentation map, respectively; ∇ represents the gradient operation of the image; and λ is the weight coefficient of the smoothing term, which is used to control the degree of influence of the boundary smoothing term in the total loss.

The final loss function combines the focal Tversky loss and the boundary smoothing term and can be expressed as follows:

(15) $$L_{total}=L_{Focal\;Tversky}+L_{smooth}$$

Through this weighted combination, our model can simultaneously focus on difficult-to-classify samples and fuzzy boundaries during the training process, thereby significantly improving the segmentation accuracy.

#### *Advantages of module combination*

In this study, we propose an innovative CSFTU-Net model that integrates the CBAM and the SE mechanisms, as well as focal Tversky loss, to address the intricacies of CT image segmentation tasks for corn endosperm. Specifically, the CBAM enhances U-Net’s feature extraction capability through saliency and channel attention mechanisms, enabling the model to focus more effectively on critical feature regions by weighting both the spatial and channel attention of the feature map, thus improving segmentation accuracy. Furthermore, the SE module optimizes feature representation through adaptive recalibration, allowing the model to dynamically adjust its attention to different features. This combination equips CSFTU-Net with the capacity to capture intricate internal structures in CT images with low contrast and blurred boundaries. Additionally, to address the blurred boundaries and class imbalance issues in maize endosperm segmentation, we employ the focal Tversky loss function and include a boundary smoothing term as an extra loss component. This approach emphasizes challenging-to-classify pixels, particularly in the context of class imbalance, by assigning greater weights to misclassified pixels, thereby directing the model’s attention toward difficult-to-segment regions during training. Our experimental findings demonstrate that when combined with focal Tversky loss and the boundary smoothing term, CSFTU-Net outperforms the traditional U-Net architecture in segmenting maize endosperm, particularly in distinguishing between vitreous and starchy endosperm parts.

Through the integration of the above technologies, the CSFTU-Net model not only improves the accuracy and robustness of CT image segmentation for maize endosperm but also lays a foundation for future applications in other crop image segmentation tasks. These innovative design ideas give our models important application potential in intelligent agriculture and precision crop trait identification.

### Extraction of the internal structure phenotype for maize kernels

**Fig. 6:**
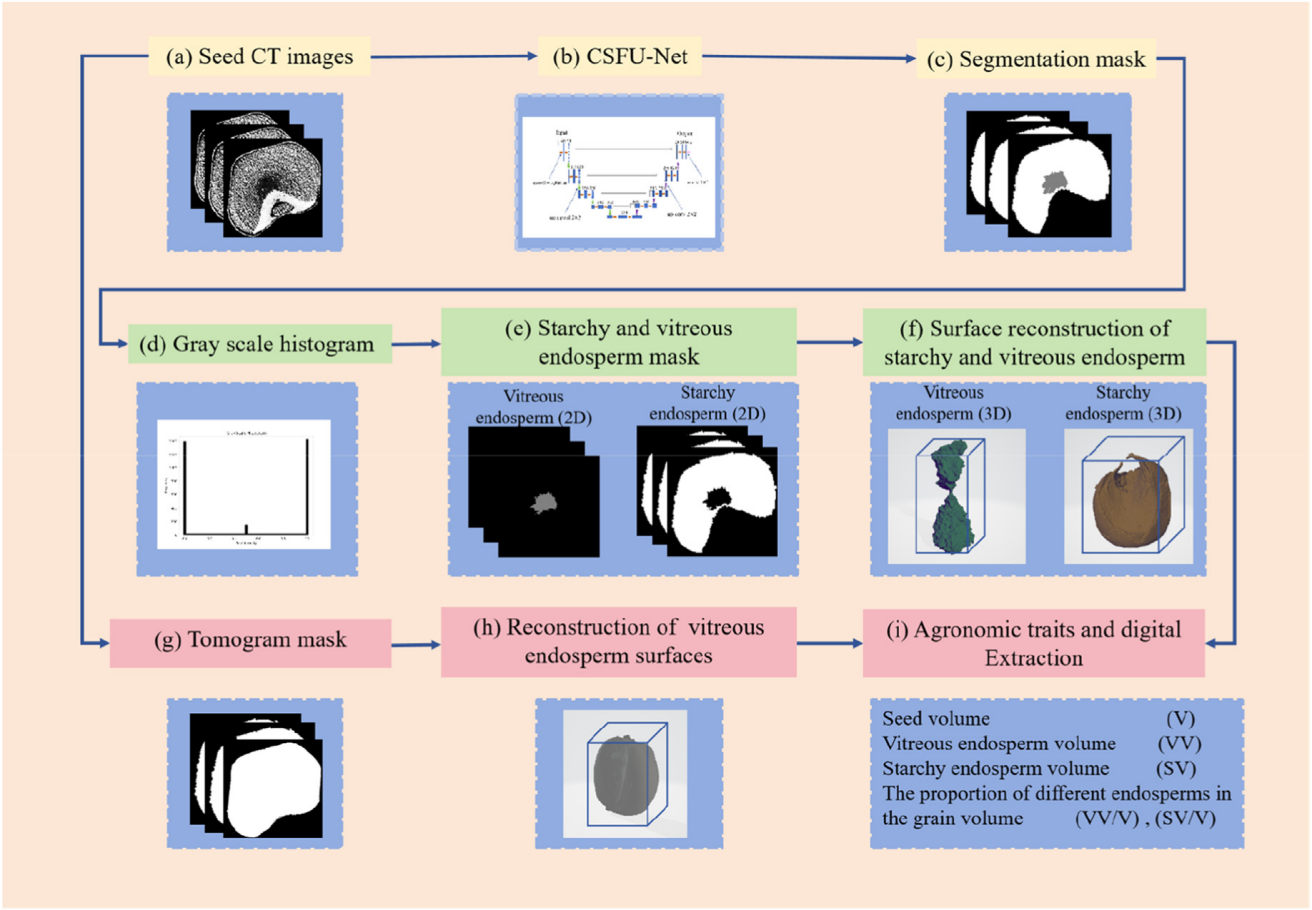
Extraction of three-dimensional traits of seeds and their vitreous and starchy endosperm. (a)–(f) 3D model reconstructions of vitreous and starchy endosperm. (a)–(h) Reconstructions of the 3D models of the grains. (i) Extraction of volume-related indicators after 3D reconstruction.

Fig. 6 shows the workflow for extracting internal components and structures from a single maize kernel. The arrows in the diagram indicate the segmentation and surface reconstruction of the vitreous endosperm, starchy endosperm and maize kernel. Initially, CT images of kernels are input into CSFTU-Net (Fig. 6(a) and (b)), which automatically segments the masks for vitreous and starchy endosperm (Fig. 6(c)). A histogram analysis is subsequently conducted on the segmented images (Fig. 6(d)). Using Otsu’s algorithm, a binarization threshold is set to separate the vitreous endosperm mask from the starchy endosperm mask, producing the final mask images (Fig. 6(e)). The image mask of seeds is extracted via Open-CV (Fig. 6(g)).

The internal structure of the maize based on the CT images has mainly a geometric shape. The volume, volume ratio, length, width and thickness of vitreous and starchy endosperm can be calculated using masked three-dimensional images. The three-dimensional surface models of vitreous and starchy endosperm (Fig. 6(f)) are the results of mask (Fig. 6(e)) reconstruction and are used to calculate the volume (Fig. 6(i)). The steps and formulas are as follows: Initially, the mask images are converted into voxel volume data by stacking the 2D mask images to construct a three-dimensional volume. The formula is as follows:

(16) V(x,y,z)=mask(x,y,z)

In this context, V(x,y,z) represents the voxel volume data, where the masks for the starchy endosperm and the floury endosperm are binary.

The volume of the reconstructed model is subsequently computed. Here, we assume that each voxel has a volume of ΔV, and the total volume is obtained by summing the contributions of all voxels. The formula is as follows:

(17) $$Volume=\sum_{x,y,z}V\left(x,y,z\right)\cdot \Delta V$$

where ΔV denotes the volume of a single voxel, which in a regular grid can be expressed as follows:

(18) $$\Delta V={Voxel\_size}^{3}$$

where Voxel_size refers to the edge length of each voxel. For this study, Voxel_size is 27 ​μm ​× ​27 ​μm ​× ​27 ​μm.

Additionally, the length, width, and thickness of the vitreous and starchy endosperm are expressed using the three edge lengths of the bounding box in the three-dimensional directions of their surface models, where the longest edge is the length, the next longest edge is the width, and the shortest edge is the thickness. Finally, the calculated results are saved to an Excel file (Fig. 6(I)). This study provides five phenotype parameters, as shown in Table 2.

**Table 2:** Phenotypes of the internal grain structure parameters.

Phenotype index	Abridge	Define
Seed volume	V	Seed surface modeling volume
Vitreous endosperm volume	VV	Model volume of the vitreous endosperm surface
Starchy endosperm volume	SV	Model volume of the starchy endosperm surface
Vitreous endosperm volume percentage	VV/V	Ratio of vitreous endosperm to seed volume
Starchy endosperm volume percentage	SV/V	Ratio of starchy endosperm to seed volume

## Experimental environment analysis of results

### Experimental environment and evaluation metrics

We use a Windows system, Python 3.9, and PyTorch 1.13.1 on an NVIDIA GeForce RTX 3060Ti system. Training and testing are performed using identical hyperparameters: 50 epochs, the RMSprop optimizer, a batch size of 1, an initial learning rate of 0.0001, and a minimum learning rate of 0.00001. The focal Tversky loss function is applied. The calculation formula is shown in Equations (12), (13).

Network accuracy is assessed using the Dice coefficient and intersection over union (IoU).

(19) $$D_{ice}=\frac{2\left|X\cap Y\right|}{\left|X\cup Y\right|}$$

(20) $$I_{ou}=\frac{\left|X\cap Y\right|}{\left|X\cup Y\right|}$$

where X denotes the set of predicted pixels, Y represents the set of real pixels, $D_{ice}$ represents the Dice coefficient, and $I_{ou}$ represents the intersection over union.

### Comparison of different segmentation models

**Fig. 7:**
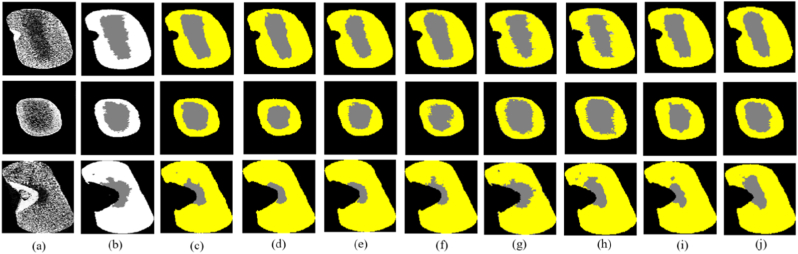
Comparison of the segmentation results of the improved model and several traditional segmentation models. (a) Original data (b) Mask (c) FCN (d) DeepLab (e) TransU-Net (f) U-Net (g) CSFTU-Net (h) TransU-Net++ (i) Swin UNETR (j) SAM-Med3D.

The segmentation results of the CSFTU-Net model are compared with those of U-net, FCN, DeepLabv3, TransUNet, TransU-Net++ [37], SwinUnetr [38] and SAM-Med3D [39] to evaluate its overall performance. In the comparative experiment, the same dataset and loss function are used for these segmentation models, and the segmentation model is tested by the evaluation index introduced in Section 4.1. The CSFTU-Net model is superior to the other models in terms of segmentation results. It can accurately capture the boundaries and details of the target and improve the segmentation accuracy and precision (Fig. 7). In addition, the CSFTU-Net model improves the processing of class imbalances, resulting in more balanced and stable segmentation results.

According to the results in Table 3, the performance of each model in vitreous and starchy endosperm segmentation tasks significantly differed. Specifically, the DeepLabv3 model performed the worst, with a Dice coefficient and an IoU of 81.53 ​% and 71.32 ​%, respectively, indicating insufficient segmentation accuracy when dealing with complex structures. Compared with DeepLabv3, the FCN model has improved, with the Dice coefficient and IoU reaching 85.02 ​% and 77.07 ​%, respectively, but it still requires further optimization to meet the needs of high-precision segmentation. The TransU-Net model performs slightly better than DeepLabv3 and U-Net do but performs worse than the FCN does. Notably, the TransUNet ++ and SAM-Med3D models perform similarly, with Dice coefficients of 86.59 ​% and 86.76 ​%, respectively, and IoUs of 72.92 ​% and 76.98 ​%, respectively, indicating high segmentation accuracy, but they still do not reach the level of CSFTU-Net. In contrast, the Swin UNETR model performed moderately, with a Dice coefficient and an IoU of 83.33 ​% and 73.54 ​%, respectively. The CSFTU-Net model performed best, with a Dice coefficient of 89.13 ​% and an IoU of 76.56 ​%, indicating that it has significant advantages in terms of segmentation accuracy and detail capture ability, especially in dealing with the complex structure of the maize endosperm. Excellent recognition and segmentation capabilities are achieved.

**Table 3:** Comparison of the accuracies of different deep learning models on the test set.

Module	Dice (%)	IoU (%)
FCN	85.02	77.07
DeepLabv3	81.53	71.32
TransU-Net	84.89	75.68
U-Net	81.08	71.21
CSFTU-Net(Ours)	89.13	76.56
TransUNet++	86.59	72.92
Swin UNETR	83.33	73.54
SAM-Med3D	86.76	76.98

### Self-comparison of CSFTU-net

We conducted a self-comparison of the CSFTU-Net model to evaluate its performance in CT image segmentation tasks for corn seeds. The experiment is divided into two parts: first, we compare the training results of the original data and the data enhanced via the U-Net model; second, we analyze the influence of different attention mechanisms on segmentation performance.

#### *Comparison of original data and enhanced data*

**Fig. 8:**
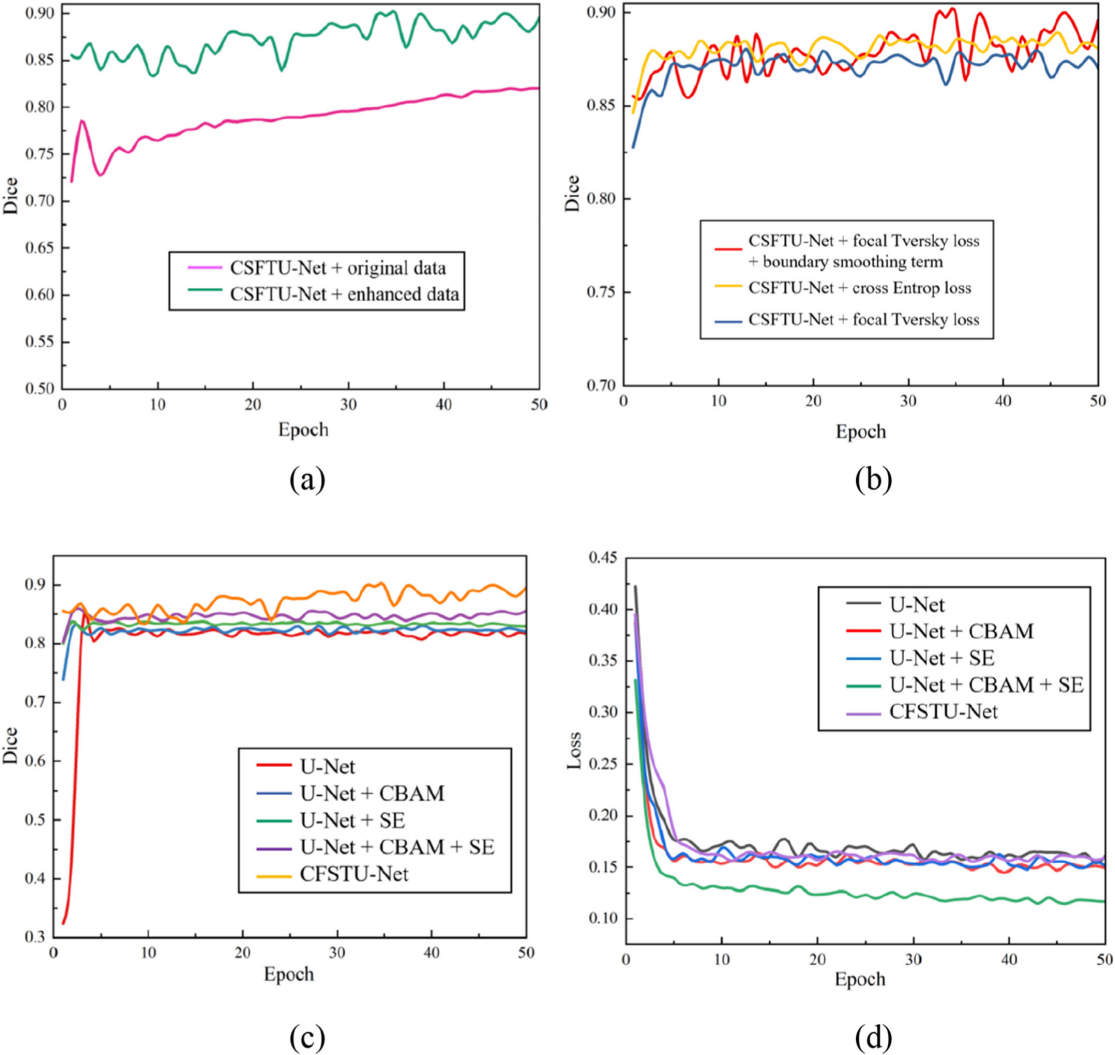
(a) Loss values and accuracy curves of the original data and the enhanced data for the U-Net model. (b)–(c) Loss values and accuracy curves for different models on the same training set. (d) Accuracy curves of different loss functions on the CSFTU-Net model.

We use the U-Net model to train the original data and the enhanced data and evaluate the effectiveness of the model through the loss value and the Dice coefficient curve (Fig. 8(a)). The results show that after data augmentation, the model has a lower loss value and a higher Dice coefficient in the training process, indicating that data augmentation significantly improves the training effect of the model. This result verifies the importance of data augmentation in improving model generalizability and segmentation accuracy.

#### *Influence of different attention mechanisms*

To further optimize the segmentation performance of the U-Net model, we explored the influence of the introduction of different attention mechanisms, including the CBAM, the SE module, and their combination (CBAM ​+SE), on performance. On this basis, we develop a model (named CSFTU-Net) by combining focal loss and Tversky loss. By comparing and analyzing the segmentation performance of each model (Fig. 8(a)-(d)), we find that the Dice coefficient of the basic U-Net model is 81.76 ​%; after introducing the CBAM into U-Net, the Dice coefficient increases to 83.31 ​%, which confirms that the CBAM can effectively increase the model’s attention to key features. Furthermore, after U-Net is combined with the SE module, the Dice coefficient significantly increases to 85.10 ​%, which highlights the significant advantages of the SE module in adaptive feature recalibration. When the CBAM and SE module work together on U-Net, the Dice coefficient of the model reaches 87.58 ​%, which further improves the segmentation accuracy. Finally, the CSFTU-Net model using focal Tversky loss achieves a Dice coefficient of 89.13 ​% because of its excellent ability to deal with unbalanced categories, indicating a significant improvement in model performance.

**Fig. 9:**
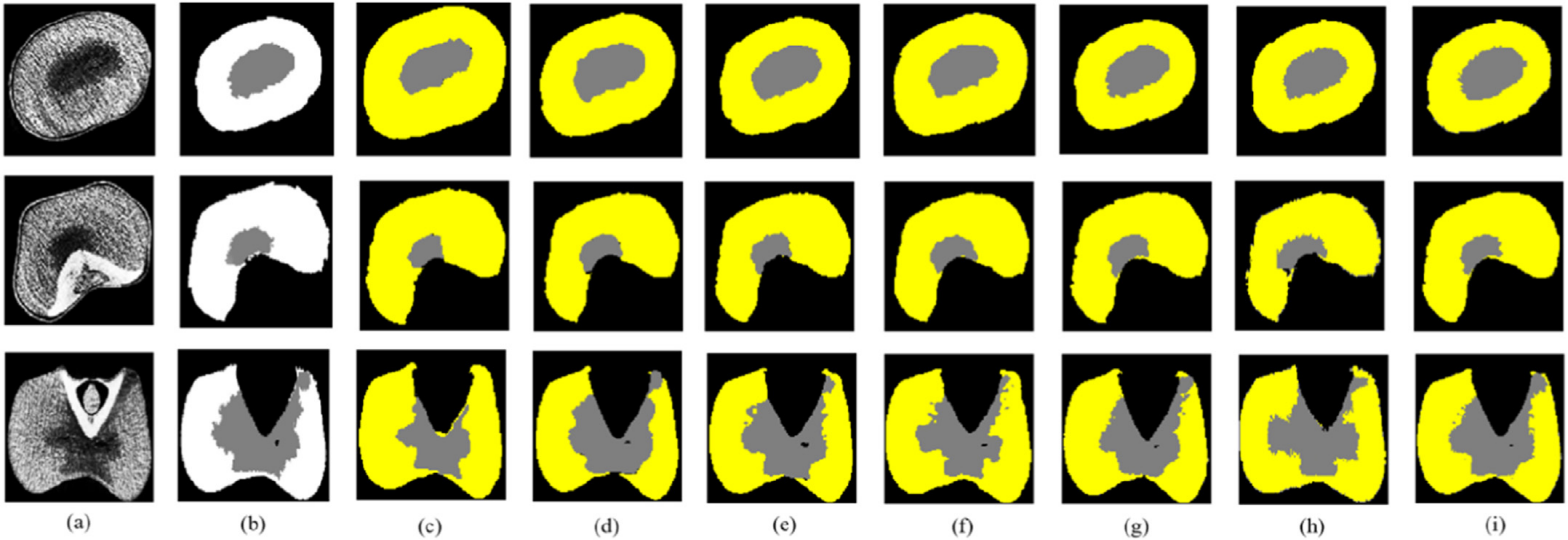
Segmentation results after adding different modules to the U-Net model. (a) Original image (b) Mask (c) U-Net ​+ ​original data (d) U-Net ​+ ​enhanced data (e) UNet ​+ ​CBAM (f) UNet ​+ ​SE (g) UNet ​+ ​CBAM ​+ ​SE (h)–(i) Comparison of different loss functions, where (h) uses the cross-entropy loss function and (i) uses the focal Tversky loss function.

In addition, the original U-Net semantic segmentation model fluctuates too much at the beginning of convergence, while the model can converge faster and achieve higher accuracy after the attention mechanism module is added. Table 4 shows that incorporating the CBAM and SE attention mechanisms enhances segmentation performance by focusing on both channel and spatial attention mechanisms while also modeling the interdependence between convolution feature channels. Second, the focal Tversky loss function assigns greater weights to misclassified pixels. This approach directs the model’s focus toward challenging pixels during training, enhancing its overall performance. As illustrated in Fig. 9, the proposed method demonstrates superior performance in edge segmentation.

**Table 4:** Comparative analysis of the segmentation performances of the U-Net models with different models.

Basic model	Original data	Enhanced data	CBAM	SE	Original Loss	Focal Tversky Loss	$L_{total}$ (Our)	Dice (%)
U-net	**✓**				**✓**			75.63
		**✓**			**✓**			81.76
		**✓**	**✓**		**✓**			83.31
		**✓**		**✓**	**✓**			85.10
		**✓**	**✓**	**✓**	**✓**			87.58
		**✓**	**✓**	**✓**		**✓**		88.05
		**✓**	**✓**	**✓**			**✓**	89.13

### Phenotype analysis of maize varieties

**Fig. 10:**
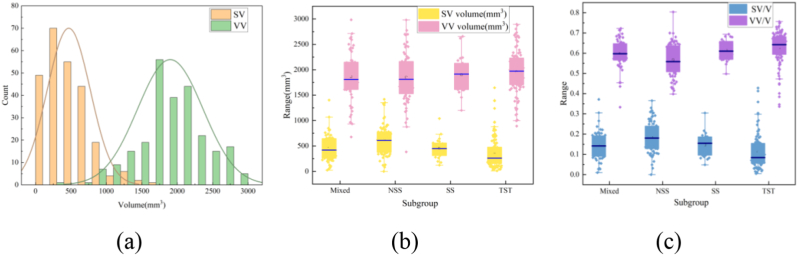
Analysis of seed endosperm data. (a) Volume distribution of vitreous and starch endosperm. (b) Volume distributions of vitreous and starch endosperm in different subpopulations. (c) Distribution of vitreous and starch endosperm volume in different subpopulations.

First, the phenotypic data for all the maize grains are statistically analyzed. The volume range for starchy endosperm is 0–1500, and the range for hard endosperm is 500–3000, as shown in Fig. 10(a). In this work, a total of 250 maize grain varieties are used, including 59 varieties in the mixed subgroup, 67 varieties in the NSS subgroup, 28 varieties in the SS subgroup and 96 varieties in the TST subgroup. Second, to investigate the discrimination of endosperm phenotypes in different subgroups of maize varieties, volume and volume ratio box plots of starchy and vitreous endosperm in different subgroups of maize varieties are drawn (Fig. 10(b)–(c)). There are significant differences in the volume and volume ratios of starchy and vitreous endosperm among the four subgroups of grains.

Understanding these proportions can aid breeders in selecting maize varieties for specific processing needs. Vitreous endosperm, which is high in amylose, offers greater mechanical strength during processing, making it ideal for cornflakes and puffed foods. Starchy endosperm, which is high in amylopectin, is softer and more prone to breakage and is better suited for cornmeal and corn oil production. This knowledge helps farmers choose varieties that meet market demands, enhancing economic returns.

## Discussion

### Complexity and fine-grained detail capture

Although the modified U-Net (CSFTU-Net) performs well in segmenting the internal structure of maize kernels, achieving a Dice score of 88.09 ​%, which is significantly better than that of the traditional model, the complexity of the internal structure of maize kernels may still have a certain impact on the model’s ability to capture fine-grained details. The structure of the maize endosperm contains not only the two main components of the vitreous and starchy endosperm but also a variety of microstructural characteristics, such as stomata, cell walls and starch granules. These subtle structural differences may manifest as similar gray values in CT images, especially in areas with blurred or overlapping boundaries, and the model may have difficulty accurately distinguishing these different types of endosperm.

In practical applications, the internal endosperm part of the maize kernel often presents a complex hierarchical structure and irregular shape, which places greater requirements on the segmentation model. Although CSFTU-Net enhances the feature extraction ability by introducing the CBAM and the SE module to increase the attention to important features, the model may still face challenges when dealing with blurred or overlapping regions. This situation may lead to a decrease in the accuracy of the segmentation results, affecting the subsequent quantitative analysis and phenotypic parameter extraction.

In addition, the CT images of maize kernels are affected by many factors, such as imaging conditions, sample selection and image preprocessing, which may lead to differences in image quality and further aggravate the difficulty of capturing fine-grained details. Therefore, future research should consider introducing more advanced image processing techniques, such as multiscale feature extraction, image enhancement and adaptive threshold segmentation, to improve the performance of the model for complex structures.

### Challenge of edge segmentation

Edge segmentation is a key problem in image segmentation, especially when dealing with objects with unclear boundaries. In this study, the internal endosperm structure of maize kernels is complex, especially at the boundary between the vitreous and starchy endosperm, and there is often ambiguity and overlap. This phenomenon makes edge segmentation particularly challenging. Although the CBAM and SE module are introduced in CSFTU-Net to enhance the attention mechanism of the model and increase the attention to important features, in some cases, the model may still be unable to capture subtle edge changes effectively.

Specifically, the difficulty of edge segmentation is reflected mainly in the following aspects. First, in the CT image of corn kernels, the contrast between the inner endosperm and the external structure may be low, which makes it difficult for the model to identify the edge accurately. Second, in complex backgrounds, the interference of other tissue structures may mask the boundary of the inner endosperm, which further increases the difficulty of segmentation. In addition, the existence of low-contrast regions makes it difficult for the model to learn clear boundary features during training, distorting the segmentation results.

To address these challenges, future research can consider combining the advantages of edge detection technology and deep learning models. For example, the use of gradient-based edge detection algorithms (such as Canny edge detection) combined with CSFTU-Net may help improve the sensitivity of the model to edges. In addition, the multiscale feature fusion method can enhance the model’s ability to capture edge information at different scales, thereby improving the segmentation accuracy.

In summary, although CSFTU-Net performs well in the segmentation of partial structures in maize kernels, it still faces challenges in edge segmentation. By further optimizing the model structure and introducing advanced edge detection technology, future research will help improve the segmentation performance of the model in complex backgrounds and low-contrast regions, thus promoting the application and development of intelligent agriculture.

### Sensitivity to noise

In practical applications, the input image may be affected by noise, especially in the processing of CT images of corn kernels, and the presence of noise may significantly interfere with the segmentation effect of the internal endosperm. Although the CSFTU-Net model optimizes the training process by introducing the focal Tversky loss function, which aims to increase the attention of the model to difficult-to-classify samples, thereby improving the segmentation performance, the robustness of the model may be insufficient in a high-noise environment, resulting in unstable segmentation results.

Specifically, noise can come from many factors, such as the limitations of imaging equipment, environmental factors or the characteristics of the sample itself. This noise may be expressed as random gray fluctuations, which reduce the overall quality of the image, making it difficult for the model to accurately identify the boundary and structural features of the endosperm during training and reasoning. In the case of considerable noise, CSFTU-Net may be affected, resulting in errors in the segmentation results and even artifacts that affect subsequent quantitative analysis and phenotypic parameter extraction.

To solve this problem, future research can consider combining image preprocessing techniques, such as noise removal and image smoothing, to improve the quality of the input image. In addition, the use of data enhancement techniques to increase the diversity of training samples, especially samples under noisy conditions, can help the model better learn robust feature representations, thereby improving its segmentation performance in noisy environments.

### Future work

We plan to further explore the potential of CSFTU-Net in future research, including optimizing the network structure and training strategy, and consider applying it to image segmentation tasks for other crops to verify its versatility.

## Conclusion

This work employs deep learning technology for nondestructive segmentation of vitreous and starch endosperm in maize kernels. Initially, we acquire sectional images of kernels using X-ray computed tomography, construct CT datasets, and perform image preprocessing. We enhance the U-Net segmentation network (CSFTU-Net) by incorporating CBAMs in each downsampling layer of the encoder and SE modules in each upsampling layer of the decoder, improving the segmentation and recognitionability to segment and recognize internal endosperm structures. We also introduce the focal Tversky loss function to optimize training strategies, enhancing overall model accuracy. Subsequently, we assess the segmentation precision. Finally, we perform a quantitative analysis of these segmentation results, quantify the volume distributions of vitreous and starch endosperm among different varieties and subgroups and conduct a phenotype analysis. These analyses not only reveal the detailed internal structures of the kernels but also provide crucial genetic information for future varietal improvements.

## Author contributions

J. Wang conceived the idea and designed the method, S. Yang and Xinyu Guo provided comments, and C. Wang contributed to the preparation of materials and equipment. J. Wang and Xinyu Guo carried out the experiments. J. Wang and S. Yang analyzed the data and interpreted the results. J. Wang wrote the paper and crafted all the figures and tables. C. Wang, Y. Zhang, W. Wen and Xinyu Guo revised the paper. All the authors read and approved the final manuscript.

## Data availability

The data used to support the findings of this study are available upon request from the corresponding author.

## Funding

This research was supported by the 10.13039/501100012166National Key Research and Development Program (2021YFD1200705), the Collaborative Innovation Center of the 10.13039/501100007934Beijing Academy of Agricultural and Forestry Sciences (KJCX20240406), and the Science and Technology Innovation Special Construction Funded Program of the 10.13039/501100007934Beijing Academy of Agriculture and Forestry Sciences (KJCX20220401).

## Declaration of competing interest

The authors declare that they have no known competing financial interests or personal relationships that could have appeared to influence the work reported in this paper.
